# Deep learning image enhancement algorithms in PET/CT imaging: a phantom and sarcoma patient radiomic evaluation

**DOI:** 10.1007/s00259-025-07149-7

**Published:** 2025-02-27

**Authors:** L. M. Bonney, G. M. Kalisvaart, F. H. P. van Velden, K. M. Bradley, A. B. Hassan, W. Grootjans, D. R. McGowan

**Affiliations:** 1 Sir William Dunn School of Pathology, University of Oxford, Oxford, UK; 2 Department of Medical Physics and Clinical Engineering, Oxford University Hospitals NHS Foundation Trust, Oxford, UK; 3 Department of Radiology, Leiden University Medical Center, Leiden, The Netherlands; 4 Wales Research and Diagnostic PET Imaging Centre, University of Cardiff, Cardiff, UK; 5 Oncology and Haematology, Oxford University Hospitals NHS Foundation Trust, Oxford, UK; 6 Department of Oncology, University of Oxford, Oxford, UK

**Keywords:** Deep learning, Image enhancement, PET/CT, Radiomics, Oncology, Sarcoma, Phantom

## Abstract

**Purpose:**

PET/CT imaging data contains a wealth of quantitative information that can provide valuable contributions to characterising tumours. A growing body of work focuses on the use of deep-learning (DL) techniques for denoising PET data. These models are clinically evaluated prior to use, however, quantitative image assessment provides potential for further evaluation. This work uses radiomic features to compare two manufacturer deep-learning (DL) image enhancement algorithms, one of which has been commercialised, against ‘gold-standard’ image reconstruction techniques in phantom data and a sarcoma patient data set (N=20).

**Methods:**

All studies in the retrospective sarcoma clinical [$^{18}$F]FDG dataset were acquired on either a GE Discovery 690 or 710 PET/CT scanner with volumes segmented by an experienced nuclear medicine radiologist. The modular heterogeneous imaging phantom used in this work was filled with [$^{18}$F]FDG, and five repeat acquisitions of the phantom were acquired on a GE Discovery 710 PET/CT scanner. The DL-enhanced images were compared to ‘gold-standard’ images the algorithms were trained to emulate and input images. The difference between image sets was tested for significance in 93 international biomarker standardisation initiative (IBSI) standardised radiomic features.

**Results:**

Comparing DL-enhanced images to the ‘gold-standard’, 4.0% and 9.7% radiomic features measured significantly different (p_critical_ < 0.0005) in the phantom and patient data respectively (averaged over the two DL algorithms). Larger differences were observed comparing DL-enhanced images to algorithm input images with 29.8% and 43.0% of radiomic features measuring significantly different in the phantom and patient data respectively (averaged over the two DL algorithms).

**Conclusion:**

DL-enhanced images were found to be similar to images generated using the ‘gold-standard’ target image reconstruction method with more than 80% of radiomic features not significantly different in all comparisons across unseen phantom and sarcoma patient data. This result offers insight into the performance of the DL algorithms, and demonstrate potential applications for DL algorithms in harmonisation for radiomics and for radiomic features in quantitative evaluation of DL algorithms.

**Supplementary Information:**

The online version contains supplementary material available at 10.1007/s00259-025-07149-7.

Introduction Medical imaging is integral to oncology patient pathways, and has the potential to provide unique large-scale quantitative information that when combined with other -omic data (e.g. histology, pathology, genomics) can better characterise disease and treatment response, enabling personalised treatment. However, despite large advances in quantitative image analysis over the past two decades routine clinical image inspection remains largely qualitative [1, 2].

**Fig. 1:**
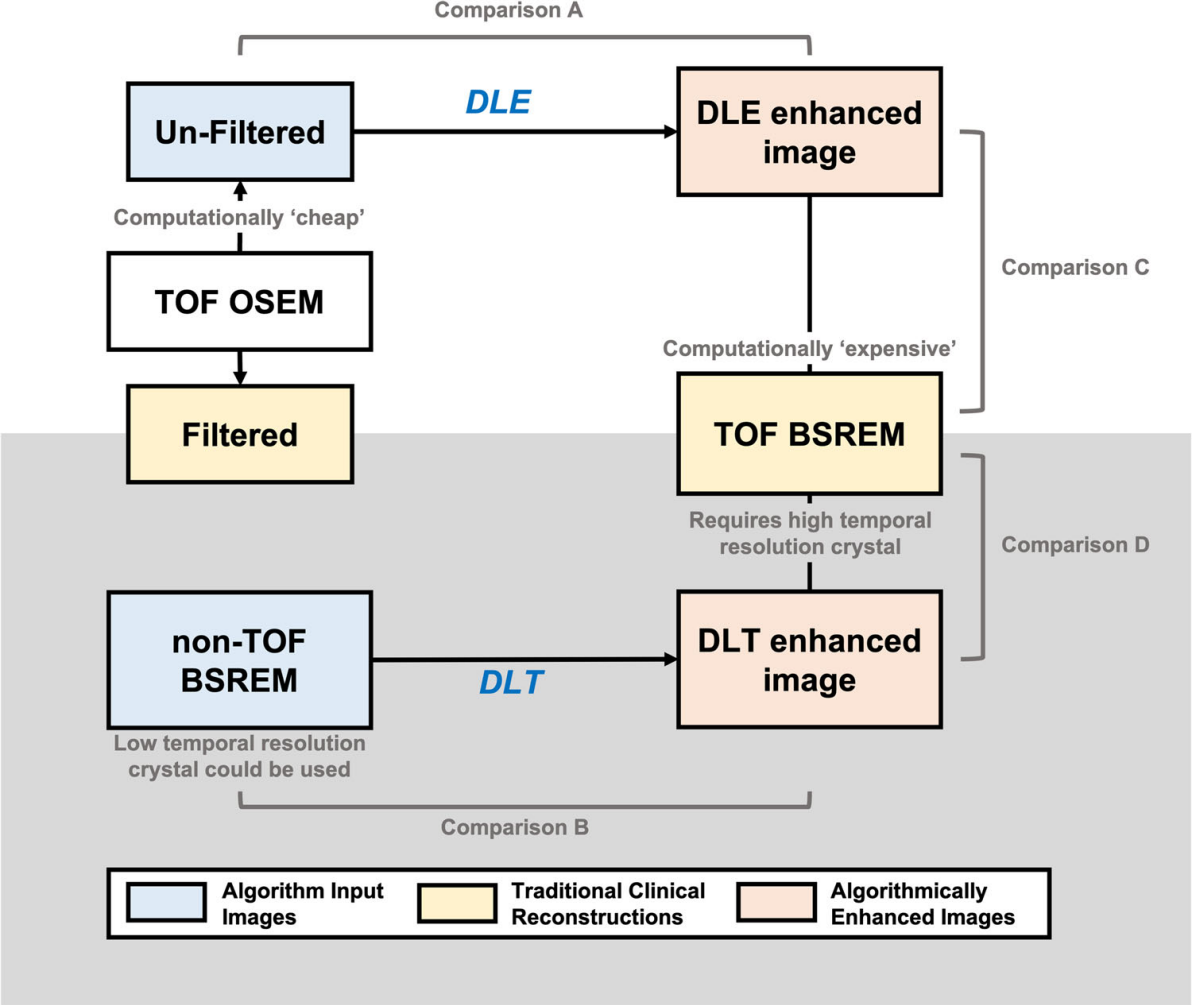
Schematic diagram of the comparisons of the purpose of different algorithms tested in this work and the comparisons performed

Medical imaging modalities vary widely in the information they provide on the underlying tissue being imaged. Particularly relevant to the field of oncology is positron emission tomography (PET). Beyond standard radiological interpretation of PET images, non-uniformity in PET image texture has been shown to be related to heterogeneity in the spatial distribution of underlying cancer cells in a tumour [3]. This is of importance as heterogeneity is known to be associated with treatment resistance and prognostic for metastatic disease, and poor clinical outcomes [4]. However, PET image texture is highly dependent on the reconstruction method deployed [5]. Recent developments in reconstruction methods include deep learning (DL) image enhancement techniques [6–8]. In this context understanding the variation of radiomic features with reconstruction method also has the potential to provide high level quantitative understanding of the DL generated images as compared to the images the algorithm was trained to emulate, serving as a secondary form of validation to clinical evaluation.

In PET imaging statistical iterative reconstruction techniques are the preferred method for most systems. Two statistical iterative techniques are used in this work. Block sequential regularisation expectation maximisation (BSREM) reconstruction, also referred to as Bayesian penalised likelihood (BPL) reconstruction, which uses a penalised likelihood reconstruction technique with a penalty term, and the more commonly used Ordered Subset Expectation Maximisation (OSEM). BSREM images are considered the ‘gold-standard’ in this work, achieving a smoother image texture and greater contrast recovery than OSEM but are computationally intensive [9]. Many DL image reconstruction techniques have been researched and developed for PET. This study focuses on two manufacturer developed algorithms. The first, deep-learning enhancement (DLE), is an algorithm trained to take an OSEM image without filter and produce a BSREM-like image, the benefit being less computational power is required than for a direct BSREM reconstruction [10, 11]. The second algorithm, deep-learning time of flight (DLT), is trained to transform a BSREM (non-time-of-flight; non-TOF) image into a BSREM (time-of-flight; TOF) image, the aim being to improve signal to noise ratio (SNR) in non-TOF data [12]. DLT has been commercialised by GE Healthcare as Precision DL (PDL). Figure 1 shows a graphical schematic of these algorithms and the comparisons which are made in this paper.

Research in the field of quantitative measurements from medical images, commonly referred to as radiomics, has been expansive, with the publication rate growing rapidly [13, 14]. Despite high prospects for radiomic analysis including promising results across numerous disease areas, systematic review papers have highlighted the limited progression of this work into clinical decision-making tools and adoption [2, 15–21]. The challenges highlighted in these papers centre on reproducing and generalising results across imaging datasets which are highly variable on many levels - patient (administered activity, respiratory motion, motion artefacts), scanner (reconstruction, noise levels, acquisition duration, scanner technology), centre (dose reference level, uptake time) [22, 23].

Phantom studies have long been used in medical imaging to characterise systems and understand the various limitations of imaging techniques including in radiomic studies [24–31]. However, no phantom can ever truly represent an in-vivo activity distribution. Hence in this work a sarcoma patients’ tumour dataset was also used for in-vivo analysis to provide verification of the phantom results in comparisons of different image reconstruction methods and enhancement techniques [10, 12].

As DL image enhancement becomes more common place, the effect on radiomic features and the implications for the generalisability of radiomic models must be considered. Where DL enhancement is used it is imperative that we understand it’s behaviour both at a visual and quantitative level. Algorithms have been clinically evaluated [10, 12, 32, 33], and changes induced in radiomic parameters investigated [34]. This work sought to compare DL-enhanced images with input images and the ‘gold-standard’ reconstruction method the algorithms are trained to emulate at a quantitative level, for two algorithms one of which is commercially available. In doing so it provides an additional validation method for DL enhancement techniques.

## Methods

### Phantom dataset

The phantom used in this study is a unique phantom designed for a multi-modality study of radiomic feature variability by Kalisvaart et al. [30]. The phantom has three inserts of different detail sizes, each with four compartments with a cubic geometry, a figure displaying the phantom components is shown in Supplementary Figure 1. The elemental cube size for the large detail size is 10.0mm, the medium insert 7.5mm and the small insert 5.0mm. The three inserts stack together to form a single cylindrical insert for the NEMA IEC image quality phantom. A more detailed description of the phantom is provided in previous work of Kalisvaart et al. [30].

The phantom was filled with total activity of 22.1 MBq of [$^{18}$F]-FDG, the activity concentration targeted was 2:4:8:16:32 kBq/ml between the five compartments (four detail compartments and background). The same as that targeted in the original work by Kalisvaart et al. [30]. The fill ratios between compartments were verified to be within 3% of the target percentage relative to the maximum for all compartments using a Wallac Wizard 2470 sample counter (Perkin Elmer) with a 20% energy window from the photopeak, detailed results provided in supplementary material (Supplementary Table 1).

Five acquisitions were acquired on a Discovery 710 (TOF) PET/CT scanner (GE Healthcare), each acquisition used two bed positions. The phantom position was varied by $\mathrm {\pm }$ 5 degrees between acquisitions in the axial, sagittal and coronal planes. The exact offsets are provided in the supplementary material (Supplementary Table 2). The acquisitions were retrospectively re-binned to achieve count statistics comparable to 32 kBq/ml in the highest activity concentration insert at 3 min per bed position. Pixel values were converted to standardised uptake value (SUV) for quantitative comparison between images, using a phantom weight of 10 kg and the total activity inserted into the phantom.

### Patient dataset

Twenty sequential retrospective sarcoma [$^{18}$F]FDG PET/CT studies, with an [$^{18}$F]FDG avid tumour volume identified in the clinical radiologist report scanned at Oxford University Hospitals were selected, study approved by the Health Research Authority (24/HRA/1339). The sample size is similar to that used in other works looking at radiomic feature robustness to reconstruction methods for PET/CT [35, 36]. The population characteristics are shown in Table 1. PET/CT images were acquired on either a D690 or D710 GE Healthcare PET/CT system (D690 N=12, D710 N=8), these two systems are fundamentally the same from an image acquisition perspective and the acquisition protocols are matched between the two scanners. The administered activity used a weight based protocol of 4MBq/kg, with scan duration 3-minutes per bed. A CT was acquired for diagnostic and attenuation correction purposes [120 kV, pitch: 0.984, automA with noise index of 25]. The standard clinical PET reconstruction parameters are shown in Table 2.

**Table 1:** Patient population characteristics, specific sarcoma diagnosis is not provided as the groups of patients are too small to retain anonymisation

Characteristic	Statistic
Age (years)	Median (IQR): 56 (41 - 63)
Sex (n)	Male: 9, Female: 11
BMI (kg/m$^{2}$)	Median (IQR): 26.5 (19.5 - 34.7)
Uptake time (minutes)	Median (IQR): 89.4 (87.3 - 94.7)
Tumour Volume (mL)	Median (IQR): 214 (104 - 631)
Tumour Location (n)	Limb: 12, Other: 8

**Table 2:** PET reconstruction parameters used in this work

Reconstruction	Parameters	
OSEM	OSEM Standard Reconstruction	TOF, 2 iterations, 24 subsets, 6.4mm Gaussian filter, standard z-axis filtering
	OSEM without filtering	TOF, 2 iterations, 24 subsets, no filter, no z-axis filtering
BSREM	BSREM (non-TOF)	non-TOF, weighting factor 400
	BSREM (TOF) (‘gold-standard’)*	TOF, weighting factor 400
DL	OSEM without filtering + DLE	Standard level model [10].
	BSREM (non-TOF) + DLT	High level model [12].

All images are reconstructed using a 256 x 256 matrix, with a 3.27mm slice thickness. *The reconstruction in clinical use at Oxford University Hospitals NHS Foundation Trust

**Fig. 2:**
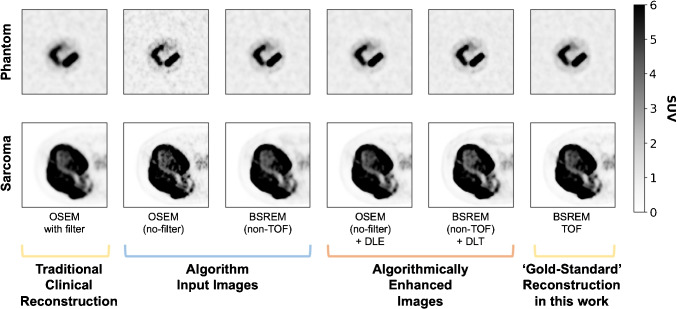
Example images from the large phantom insert and an example sarcoma tumour from the dataset (Iliac Ewing Sarcoma, axial orientation) for each of the six reconstruction and enhancement techniques used throughout this work. Images and windowing have been selected to provide a visual demonstration of differences in image texture between the reconstruction and enhancement techniques included in this work

### Reconstruction and image enhancement

As outlined in the introduction this work assesses two manufacturer developed DL algorithms, DLE and DLT. The overarching purpose of these algorithms is to take a ’lower quality’ image input and generate a ’higher quality’ output, targeting the ‘gold-standard’ reconstruction method. To describe and understand the impact of these algorithms on radiomic features, six images were generated for each repeat phantom acquisition dataset (5 $\mathrm {\times }$ 6, 30 images) and patient study (20 $\mathrm {\times }$ 6, 120 images). The parameters used to generate each image are outlined in Table 2, and the schematic diagram shown in Fig. 1 demonstrates the relation between the images. Alongside the algorithm input and output images, and the BSREM (TOF) ‘gold-standard’ reconstruction, a clinical standard filtered OSEM image was included for comparison. All images are reconstructed using a 256 x 256 matrix (2.73mm x 2.73mm pixel size), with a 3.27mm slice thickness. Example images from the phantom large insert and a sarcoma tumour are shown in Fig. 2.

### Image analysis and feature extraction

The images were resampled using linear interpolation in pyradiomics (version 3.0.1a3) [37] from 2.73 x 2.73 x 3.27mm$^{3}$ to an isotropic voxel size of 3.27 x 3.27 x 3.27mm$^{3}$. The clinical pixel size used at OUH is 2.73 x 2.73 x 3.27 mm, with an isotropic voxel size required for radiomic feature extraction, to avoid introducing noise into the image the decision was made to resample at the largest voxel dimension.

Segmentation for the phantom was generated by defining a cylindrical shaped volume of interest of fixed dimensions (Ø 60 mm and height 50 mm) for each detail size for the reference acquisition in Hermes Affinity (V3.0.5, Hermes Medical Solutions). All phantom images were registered to a randomly selected reference acquisition, to enable direct comparison of radiomic features. A rigid linear registration transform was performed in Python using the computed tomography attenuation correction (CTAC) images, with the registration then transferred to all PET images. Exact registration parameters are provided in supplementary material Table 1. An experienced Nuclear Medicine radiologist (21 years experience as a consultant) used the same software to segment tumour volumes in the sarcoma dataset.

The segmentation masks were then exported and analysed in Python using pyradiomics for feature extraction (version 3.0.1a3) [37]. The use of a fixed bin width is appropriate as PET/CT is a quantitative modality and this can aid interpretability of features [38]. Bin width is known to influence radiomic feature extraction, a single fixed bin width of 0.4 SUV was used in this work to enable isolation of the DL algorithm as the changing parameter. A bin width of 0.4 SUV was chosen to give approximately 64 bins across the full range of SUV values observed in the clinical dataset and has also been used in radiomic studies in literature [39–41]. Normalisation was not enabled to retain interpretability [23].

This work used the International Biomarker Standardisation Initiative (IBSI) standardised features [22], which do not include convolutional filter features. Identical region definition was used for each image set (in both phantom and patient data) and therefore shape features were excluded from analysis (14 features). First order features were calculated over the total volume, GLCM and GLRLM features were averaged in 3D and GLSZM, GLDM, and NGTDM features were calculated from a single 3D matrix. 93 IBSI compliant features remained with 18 first order, 24 gray-level co-occurrence matrix (GLCM), 16 gray-level run-length matrix (GLRLM), 16 gray-level size zone matrix (GLSZM), 14 gray-level dependence matrix (GLDM) and five neighbouring gray-tone difference matrix (NGTDM) features [22].

### Statistical analysis

### Inter-Reconstruction Variability

The effect of DL methods on images was assessed between four image pairings. In both phantom and tumour data the percentage difference was calculated relative to the first image listed for each comparison. The first two comparisons (A and B) looked to compare the effect of DL enhancement on the image the enhancement was applied to (input image). The second two comparisons (C and D) looked to compare the DL-enhanced image with the BSREM (TOF) image (‘gold-standard’) the algorithm is trained to emulate. Figure 1 provides a schematic diagram of the comparisons.

A. OSEM (TOF) without filtering vs. OSEM (TOF) without filtering + DLE
B. BSREM (non-TOF) vs. BSREM (non-TOF) + DLT
C. BSREM (TOF) (‘gold-standard’) vs. OSEM (TOF) without filtering + DLE
D. BSREM (TOF) (‘gold-standard’) vs. BSREM (non-TOF) + DLT

#### Phantom dataset

The percentage difference between the mean feature value across the five repeats was calculated for each comparison. The significance of the difference between the five measurements was also tested. A two-sample paired t-test was performed to compare between images due to the related nature of the datasets, under the null hypothesis that the two distributions are the same. A Bonferroni correction was applied for each volume size across all features (n=93, p=0.05) giving p$_{\text {critical}}$=0.0005.

#### Patient dataset

In the tumour dataset the radiomic feature measurements are expected to be different between patients due to different underlying physiological distributions. Therefore, for each patient in each comparison the percentage difference for each feature between the two relevant images was calculated, and then the mean taken over all the percentage difference for each patient. Instead of comparing the feature value distributions directly, the distribution of percentage difference between the two images in each comparison was considered and tested for the difference from zero, under the null hypothesis that the two image datasets were the same. A Bonferroni correction was applied as in the phantom dataset (n=93, p$_{\text {critical}}$=0.0005).

## Results

### Inter-reconstruction variability

**Fig. 3:**
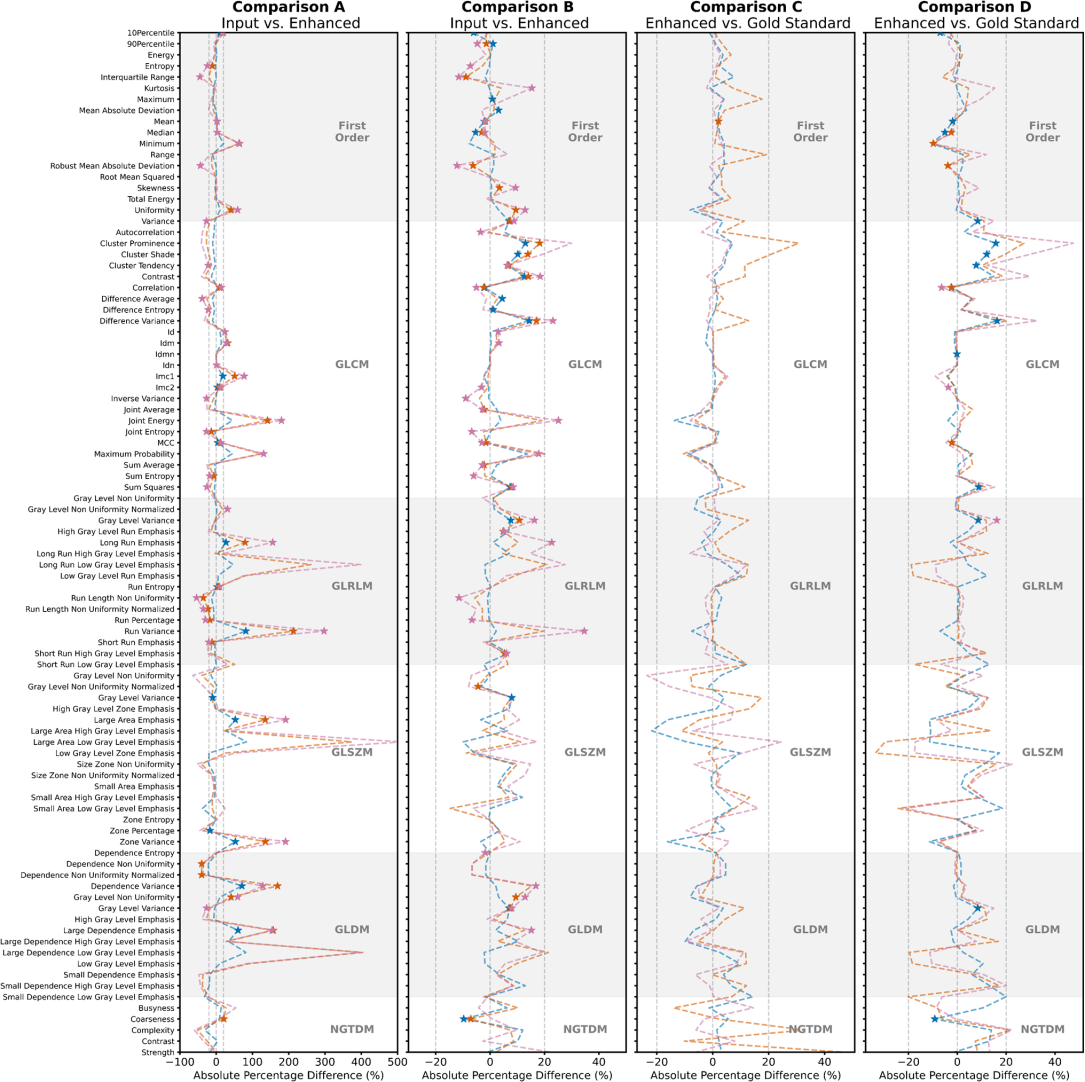
Comparisons in phantom data for three detail sizes. Starred datapoints are those for which there is a significant difference in the feature measurement at the p_critical_=0.0005 level. Comparison A: DLE enhanced image vs. OSEM input image (without filtering). Comparison B: DLT enhanced image vs. BSREM (non-TOF) input image. Comparison C: DLE enhanced image vs. BSREM (TOF). Comparison D: DLT enhanced image vs. BSREM (TOF). Dashed lines are shown at $\mathrm {\pm }$ 20% in each panel to enable comparison of effect size which varies widely

**Fig. 4:**
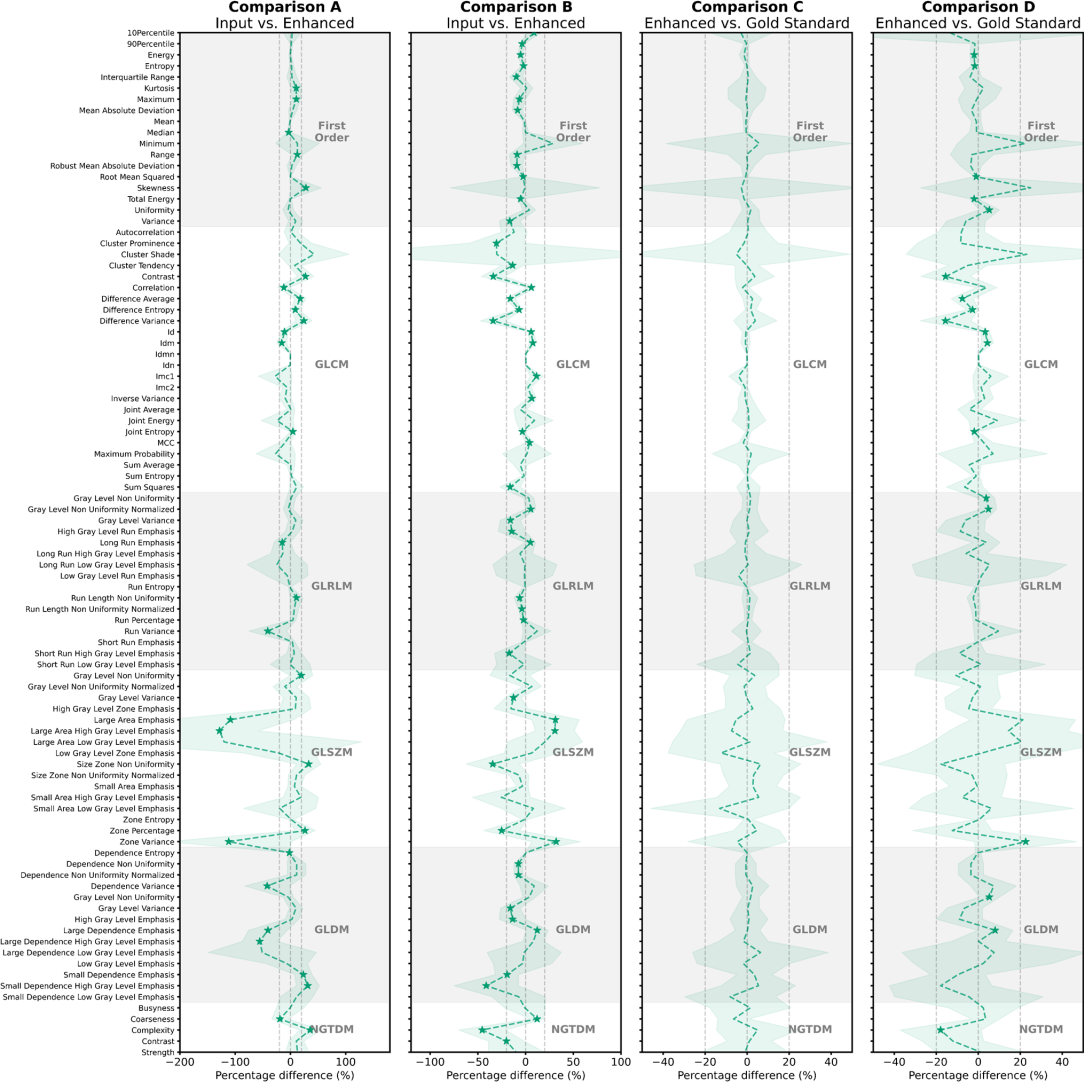
Comparisons in sarcoma patients’ tumour data (N=20). Starred datapoints are those for which there is a significant difference in the feature measurement at the p_critical_=0.0005 level. Comparison A: DLE enhanced image vs. OSEM input image (without filtering). Comparison B: DLT enhanced image vs. BSREM (non-TOF) input image. Comparison C: DLE enhanced image vs. BSREM (TOF). Comparison D: DLT enhanced image vs. BSREM (TOF). The shaded area represents ± 1 standard deviation. Dashed lines are shown at $\mathrm {\pm }$ 20% in each panel to enable comparison of effect size, which varies widely

In the phantom data variability is displayed between different features across all comparisons, as shown by the variation in signal in Fig. 3. Similar patterns of variation are seen in the sarcoma patients’ tumour data in Fig. 4. There were no discernible patterns observed between feature groups (First Order, GLCM, GLRLM, GLSZM, GLDM, NGTDM). Although, it is noted that GLRLM, GLSZM, GLDM groups displayed some more extreme feature values particularly in comparison A in the small and medium phantom details.

**Fig. 5:**
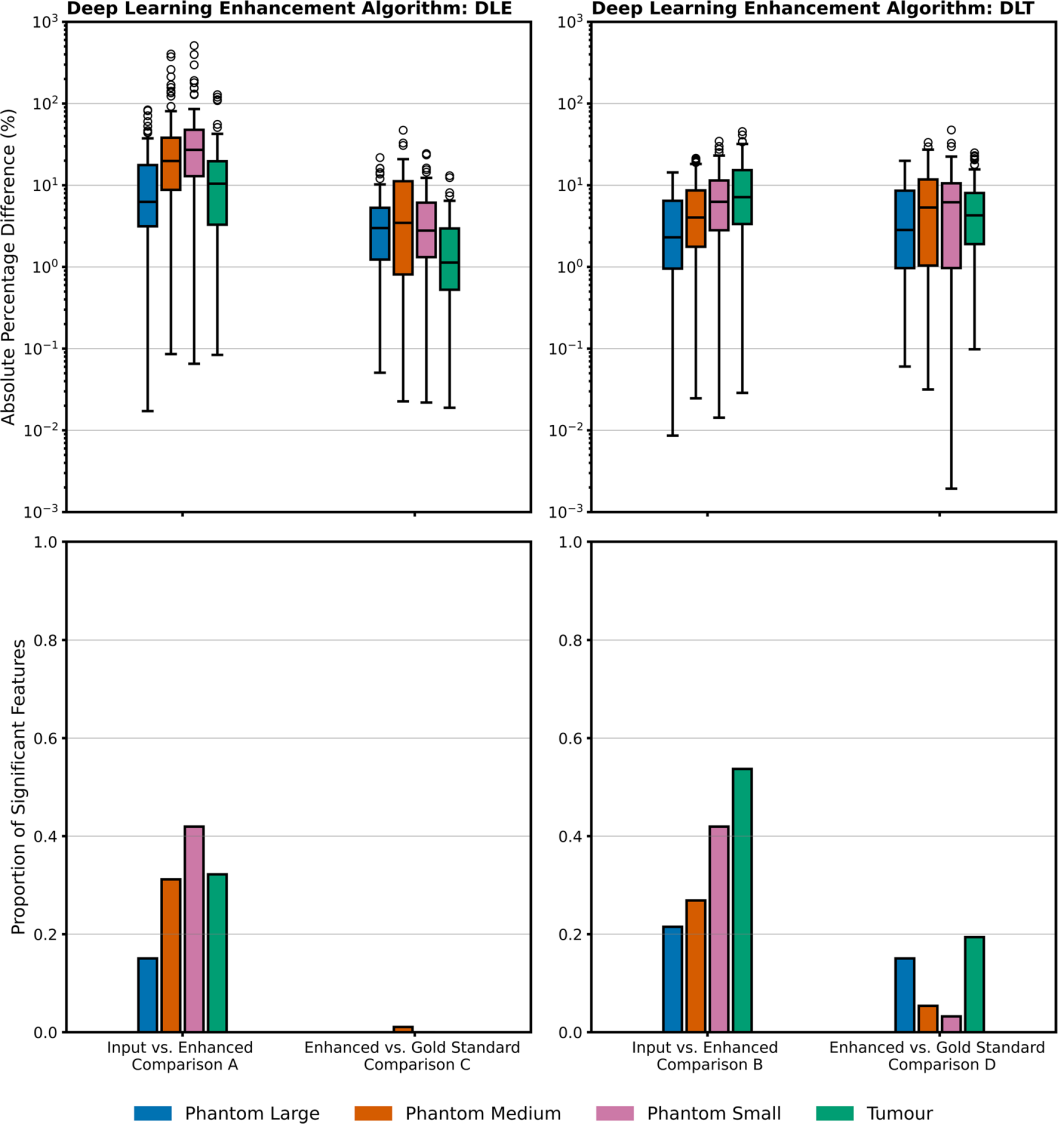
Summary data across the four comparisons for phantom and sarcoma patients’ tumour data across the distribution of 93 radiomic features. The top left panel shows the effect size is larger in Comparison A with more comparable results across the other three comparisons. In the bottom panels across all phantom volume sizes and the tumour dataset a smaller proportion of radiomic features measured as significantly different in the enhanced vs. gold standard comparisons as compared to the input vs. enhanced comparisons

Throughout all comparisons similar trends were observed in the phantom and tumour datasets. A larger proportion of features measured as significantly different in Comparison A (phantom average: 29.3%, tumour: 32.2%) and B (phantom average: 30.3%, tumour: 53.7%) than in Comparison C (phantom average: 0.4%, tumour: 0.0%) and D (phantom average: 7.6%, tumour: 19.4%), this is visualised in the lower panel of Fig. 5 for all phantom detail sizes. Notably the proportion of features that measure as significantly different decreased between Comparison A and B to Comparison C and D respectively across all datasets.

A general trend towards a larger effect size (higher average percentage difference) in Comparison A as compared to other comparisons was observed, seen in the upper panel of Fig. 5. This was found to be significantly different at the p=0.05 level from the distribution of percentage differences when comparing respective volumes (small, medium, large, tumour) from all other comparisons. The results were more mixed when comparing Comparisons B, C and D, with no clear trend. Examining the phantom data displayed in the upper panel of Fig. 5: an increasing trend in absolute percentage difference with decreasing volume size is observed in Comparison A, B and D. This trend is not observed in Comparison C. This observation cannot be made in the tumour data as there is no ground-truth knowledge of the underlying spatial structure.

While it is possible to stratify tumours by total volume, this does not necessarily provide information on the spatial scale of heterogeneity visualised within the tumour through the PET/CT image. The dependence of the effect size (percentage difference) on total tumour volume was investigated, through measuring the correlation between total tumour volume and the percentage difference in the radiomic feature measurement for each comparison. Across the four comparisons only six radiomic features returned correlations with a magnitude greater than 0.5 in more than one comparison (all six in only two of the comparisons), further exploration of these results is shown in Supplementary Figure 4.

## Discussion

The comparison of the reconstruction and enhancement techniques showed that at the quantitative level of radiomic features DL-enhanced images are similar to the ‘gold-standard’ images the DL algorithms are trained to emulate (in all data across Comparison C and D greater than 80% of radiomic features were not significantly different) but behave differently to the input images. This finding confirms the expected result based on the DL algorithm training data and demonstrates the utility of radiomic features as an end point for DL algorithm evaluation.

### Inter-reconstruction variability

Throughout all comparisons similar trends were observed in the phantom and tumour datasets, which is of note. Comparison A and B demonstrated that the DL enhancement algorithms significantly change the quantitative characteristics of the image, as measured for a large proportion of radiomic features. The greatest comparative difference was found between the OSEM without filtering image and the OSEM without filtering + DLE image (Comparison A). DLE is trained to emulate BSREM images, which are known to be significantly different visually and quantitatively from OSEM images [9]. Comparison B demonstrated that radiomic features were comparably more stable in response to the DLT algorithm, which is trained to reproduce TOF like BSREM images from a non-TOF BSREM reconstruction. The difference between comparisons aligns with the greater visual and quantitative differences expected between OSEM and BSREM images than (non-TOF) and (TOF) images of the same reconstruction method. This reiterates the importance of understanding radiomic feature dependence on image reconstruction methods, as has been highlighted in many phantom and patient datasets previously [24–30].

Quantitative analysis of PET/CT imaging for multi-site and multi-protocol data requires careful standardisation, whether the work considered uses only basic SUV parameters or more complex radiomic feature measurements. With increased commercial availability of DL image enhancement algorithms these must be considered. Harmonisation of imaging data for radiomics remains an ongoing challenge. Traditional harmonisation methods focus on accrediting centres who have achieved a set standard of SUV based quantification using phantom images, for example European Association of Nuclear Medicine Research Ltd. (EARL) [42]. This approach produces a standardised image for quantification purposes and has been shown to lead to more repeatable and reproducible radiomic feature measurements, however, it is often not the optimal image quality for lesion identification [31]. The similarity between the DL enhanced images and gold-standard images in this study demonstrate that DL image enhancement techniques could provide an alternative method for image harmonisation. This is an area of active research and will continue to be explored. The same methodology used here could be deployed in multicentre data to strengthen the conclusions.

The results also confirmed the utility of phantoms for evaluation with similar results and trends observed between phantom and patient data throughout. Phantoms enable efficient assessment of system performance, but are only useful when it is known that the phantom provides sufficient characterisation of the clinical task to be undertaken. The use of phantoms in radiomics has been limited by the relatively simple designs often used which fail to represent complex heterogeneity. This work demonstrates the ability of a heterogeneous phantom to demonstrate similar performance to clinical patient images, while also enabling a detailed assessment of system performance including for different heterogeneity detail sizes which cannot be performed in tumours with unknown ground truth size.

Large outlying differences were observed for some GLRLM, GLSZM and GLDM features in Comparison A, the images were checked and no significant artefacts are present. The outlying difference is only present in the small and medium features and is likely attributable to the different image texture generated for an OSEM type reconstruction, where all other images are ’BSREM like’. The trend towards an increased effect in decreased detail sizes in the phantom data in Comparisons A, B and D is also suggestive of a noise dominance in images of smaller detail size. Interestingly this trend was not observed in Comparison C where the lack of significantly different radiomic features suggests the images are very similar and noise profiles also likely to be similar.

Comparison C demonstrated that the DLE enhanced images were not significantly different from the BSREM (TOF) images (aside from one feature measurement), the images the algorithm is trained to emulate. DLE or DLT were trained on in-vivo imaging data, and as such the performance in phantom images, which contain angular non-anthropomorphic features is particularly notable. The two algorithms were, however, trained on different datasets. The difference in training data could explain why in Comparison D a higher proportion of radiomic features were significantly different between the DLT enhanced images and BSREM (TOF). The DLT algorithm was trained on a dataset that included imaging data from newer more advanced systems with better TOF timing resolution, (GE Healthcare DMI scanner TOF resolution 385 ps [12]) than the D710 PET/CT systems (TOF resolution 550 ps [12]). As such it is possible that the DLT algorithm has enhanced the BSREM (non-TOF) images ‘beyond’ what is achieved with a BSREM (TOF) reconstruction from the raw D710 data used in this work, this is similar to observations by Dedja et al. in the sequential application of DLT [11]. The features measured as significantly different were concentrated in first-order and GLCM features. There is minimal visual difference between images and the SUV$_{\text {MAX}}$ was not significantly different in any dataset. While it is challenging to draw conclusions from specific features, notably the measured percentage difference was negative for a number of these features, particularly in the tumour data. For example, GLCM contrast was an average of 15.8% higher (measurement of $-$15.8%) in the DLT enhanced images, with contrast increased in 18 out of 20 patient cases. This aligns with the idea that the images were enhanced ’beyond’ the D710 data.

While the decreasing trends in significant features between comparisons remain valid, the conservative nature of the Bonferroni correction may understate the total number of significantly different features. Less stringent correction methods could potentially identify additional significant features while maintaining statistical validity. However, this would not affect the key finding of this work that the enhanced images are more similar (less significantly different features) to the ‘gold-standard’ images the algorithms are trained to emulate than the input images.

This work was limited to a single site and acquisition protocol, while this enabled the isolation of the effect of DL-enhancement techniques as opposed to other induced variability, the study conclusions could be strengthened through the inclusion of multi-site data. The next phase of this work is to extend to multi-site data and different scanners/manufacturers to better understand the behaviour and stability of radiomic features. The patient population in this study was limited to 20 sarcoma patients, with a high average total tumour volume. While the dataset included a wide range of tumour volumes (Table 1), incorporating diverse disease pathologies and patient groups could further enhance the robustness and generalisability of the results. The findings from the different phantom detail sizes suggest results are dependent on the size of the underlying structure being imaged. Further investigation into the volume dependence through establishing a ground-truth comparison for the phantom could aid understanding. The development of a ground-truth would also enable a benchmark against which reconstructions could be compared and could improve understanding of the relationship between image noise and radiomic features.

## Conclusion

Previous work on deep-learning image enhancement algorithms has looked at basic quantitative metrics, but no work in literature has used radiomic parameters for in-depth quantitative assessment of the input and generated images. This paper sought to characterise the effect of state-of-the-art DL image enhancement techniques on radiomic features in patient and phantom data. In doing so this work also showed that DL generated images behave similarly, at a quantitative level, to those images the algorithms are trained to emulate, in previously unseen data.

In achieving this result we demonstrate the value of radiomic features for validation of deep learning image enhancement algorithms, where a ‘gold-standard’ exists for comparison. For a quantitative imaging modality such as PET/CT, this is an important result. Furthermore, it demonstrates the potential future scope for DL to harmonise radiomic images to ‘gold-standard’ images, aiding consistency in radiomic analysis. However, it is recognised that radiomic features are likely primarily limited by inter manufacturer variability, which is unlikely to be fully solved by an algorithm of this type.

## Supplementary Information

Below is the link to the electronic supplementary material.

Supplementary file 1 (pdf 2533 KB)

## Data Availability

Data is available under reasonable request to the corresponding author.
