# Supplementary material for: Deep learning image enhancement algorithms in PET/CT imaging: a phantom and sarcoma patient radiomic evaluation
Source: Eur J Nucl Med Mol Imaging. 2025 Feb 27;52(9):3266–77. doi: 10.1007/s00259-025-07149-7 (PMC12222275; doi:10.1007/s00259-025-07149-7)
Supplement: Supplementary file 1 — (pdf 2533 KB) [file 259_2025_7149_MOESM1_ESM.pdf]

Deep learning image enhancement algorithms in PET/CT imaging: a phantom and sarcoma patient radiomic evaluation

Supplementary Materials

1 Methods

First order features were calculated over the entire outlined volume, GLCM and GLRLM matrices were averaged in 3D, and GLSZM, GLDM and NGTDM features were calculated from a single 3D matrix. Symmetrical co-occurrence matrices and a Chebyshev norm with distance 1 were used for relevant feature classes, no distance weighting was performed.

The imaging biomarker standardisation initiative (IBSI) recommendations have been followed in this work [1]. The Python analysis pipeline was validated against the IBSI digital phantom and the pyradiomics submitted results were replicated for the feature set defined above. Only those features which conform to the standard are used in this work, except for kurtosis which is reproduced as calculated in pyradiomics with an absolute offset of three from the IBSI standardised value.

Supplementary Figure 1 shows the different components of the phantom. The insert shown in the photograph is compatible with the NEMA NU2-2018 body phantom.

**Region**	**Target Activity Concentration (kBq/ml)**	**Target Proportion of Maximum Activity Conc. (%)**	**Achieved Proportion of Maximum Activity Conc. (%)**
**Background**	32.0	-	-
**Cylinder**	16.0	50.0%	48.7%
**L-shape**	8.0	25.0%	22.7%
**T-shape**	4.0	12.5%	10.7%
**U-shape**	2.0	6.25%	5.1%

Table 1: Target vs. Achieved phantom fill activity concentration ratios

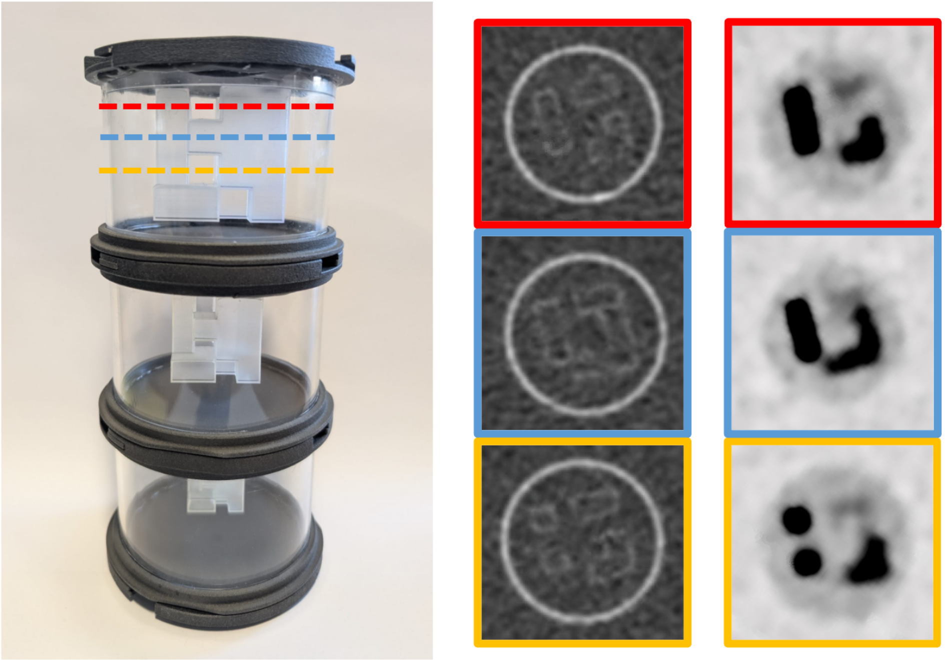

Figure 1: Photograph of the heterogeneity radiomics phantom insert, with CT slices shown from the large detail size. Images were acquired of the phantom was filled with F-18 and thus low CT contrast is achieved, the PET is windowed 0 - 6 SUV. The same geometry is repeated in the medium and small detail size inserts, shown in the lower compartments of the column.

Acquisition	Translation [x, y, z] (mm)	Rotation [C, S, A] (°)
**1**	[-2, 2, 0]	[0.0, 0.0, 3.0]
**2 - reference**	-	-
**3**	[-1, 2, -2]	[0.0, -3.5, 0.0]
**4**	[-2, 5, -4]	[0.0, 4.5, 1.0]
**5**	[2, 2, 0]	[0.0, 0.0, -2.5]

Table 2: Target vs. achieved phantom fill activity concentration ratios.

1.1 Intra-Reconstruction Variability

Additional robustness analysis was performed to enable comparison with work in literature. The intra-reconstruction variability was assessed for each feature for each detail size in the phantom data. The variability was expressed as the coefficient of quartile variation, CQV, where Q_1 is the first quartile and Q_3 is the third quartile.

$$CQV = \frac{Q_3 - Q_1}{Q_3 + Q_1} * 100 \tag{1}$$

1.2 Feature Robustness

Finally, the coefficient of variation, COV, for each feature and detail size across the four image sets that are of clinical standard (standard TOF OSEM, DLE enhanced image, DLT enhanced image and TOF BSREM), giving a total of 20 images (four reconstruction methods each with five repeats) was calculated to give a measure of overall feature robustness. The COV was used to enable comparison with literature. This could not be assessed in the tumour dataset as repeat measurements are required.

$$COV = \frac{Mean}{Standard\ Deviation} * 100 \tag{2}$$

1.3 Tumour Volume Dependence

The dependence of percentage difference in radiomic features between images for each comparison was tested for volume dependence through calculating the Pearson correlation coefficient between tumour volume and radiomic feature percentage difference across the patient cohort.

2 Results

2.1 Intra-Reconstruction Variability

The coefficient of quartile variance (CQV) was calculated for each volume size, across the five repeat acquisitions for each image reconstruction method to assess intra-reconstruction variability. Image features were grouped by feature class for the purpose of visualising variation in a heatmap (Figure 2).

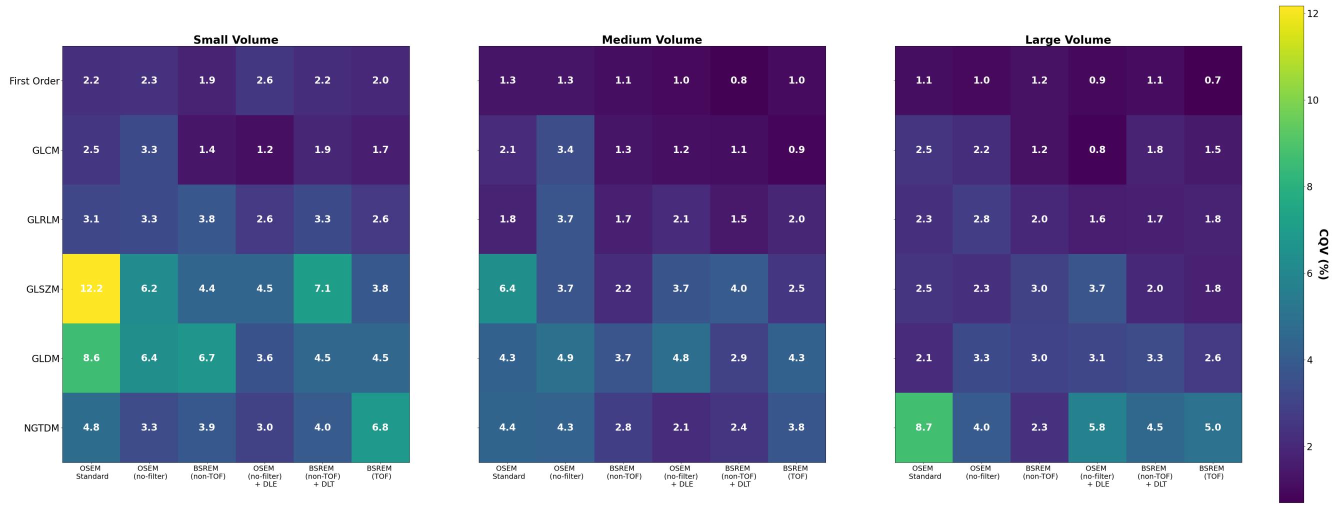

Figure 2: Intra-reconstruction variability over different feature orders for each detail size in the phantom dataset. The first column of each panel is a standard OSEM reconstruction, the next two columns are the input images to the deep-learning enhancement algorithms, followed by the two enhanced images, with the final column BSREM (TOF) considered the gold-standard reconstruction method in this work. A general trend towards OSEM reconstructions demonstrating higher intra-reconstruction variability for higher-order textural radiomic features is displayed.

2.2 Feature Robustness

The COV across all 30 images was calculated for each radiomic feature for each volume size. Taking a threshold COV of 10% to define stability, 33.3% (31/93) of features were found to be stable across all reconstruction methods (large: 44.1% (41/93), medium: 46.2% (43/93), small: 41.9% (39/93)).

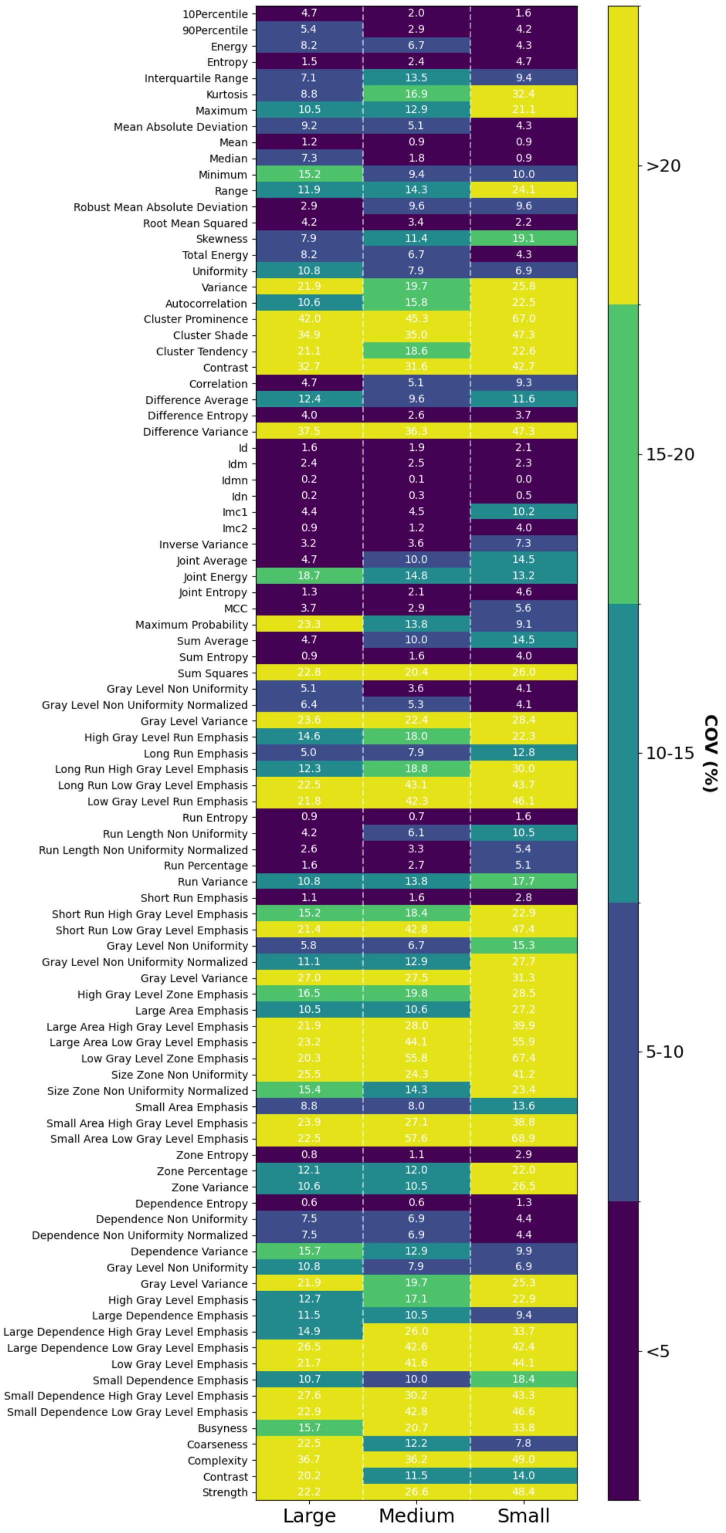

Figure 3: Coefficient of Variation for each feature across clinical standard images reconstructed (20 images: 5 repeat acquisitions, 4 reconstructions (OSEM-TOF with filter, BSREM-TOF, DLE enhanced image, DLT enhanced image)). A general trend towards higher COV in the smaller volume is seen as may be expected due to the comparable size of the detail and resolution of a PET system.

2.3 Tumour Volume Dependence

The Pearson correlation coefficient for the radiomic feature percentage difference against overall tumour volume was calculated for each comparison. The distribution was close to zero centred for all comparisons, Figure 4. Notably only six features measured as having a correlation coefficient magnitude of greater than 0.5 in more than one comparison, demonstrating no significant volume dependence.

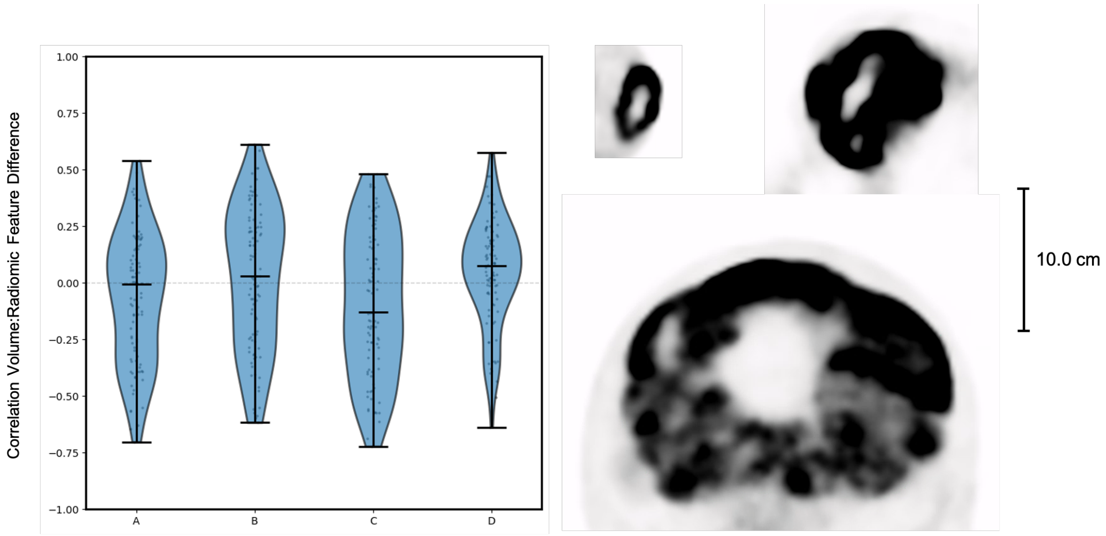

Figure 4: Violin plot of Pearson correlation between total tumour volume and the percentage difference measured between radiomic features in each comparison (A-D). Three example sarcoma tumours of varied volume (6300, 770, 80ml) are shown in the right hand side of the Figure for visual demonstration of the variability of spatial heterogeneity.

3 Discussion

3.1 Intra-Reconstruction Variability

A trend towards increased CQV for decreasing volume size is observed, with increased CQV also observed for OSEM reconstructions at higher order feature classes. It is thought this is likely due to the higher levels of background noise in OSEM reconstruction as compared to BSREM and BSREM-like reconstructions, and that for the small volume size the profile of this noise is in places comparable at a textural level to the feature details. This is in line with findings in literature, such as previous phantom work by Pfaehler et al. which demonstrated increased radiomic feature repeatability in larger volume sizes [2].

3.2 Feature Robustness

Coefficient of Variation analysis shows that 31/93 radiomic features (33.3%) are stable over all reconstruction types for all volume sizes (COV $<$ 10%). This is a comparable proportion of stable features to that observed in other phantom studies of PET/CT reconstruction method

dependence on radiomic features [3, 4]. It is important to note the four image sets included in the COV analysis generate images of a clinical standard as assessed by image quality. This is in contrast to many radiomics phantom studies which test the radiomic feature robustness using reconstruction parameters that are outside the bounds of normal clinical reconstructions.

3.3 Tumour Volume Dependence

While many radiomic features are inherently dependent on overall volume, it is expected that when considering the difference in a radiomic feature value between two images of the same underlying spatial distribution the result should not be volume dependent. The results displayed in Figure 4 align with this expectation.

References

[1] Alex Zwanenburg, Martin Vallières, Mahmoud A. Abdalah, et al. "The Image Biomarker Standardization Initiative: Standardized Quantitative Radiomics for High-Throughput Image-based Phenotyping". In: *Radiology* 295.2 (2020). PMID: 32154773, pp. 328–338.

[2] Elisabeth Pfaehler, Roelof J. Beukinga, Johan R. de Jong, et al. "Repeatability of 18F-FDG PET radiomic features: A phantom study to explore sensitivity to image reconstruction settings, noise, and delineation method". In: *Medical Physics* 46 (2 Feb. 2019), pp. 665–678. ISSN: 24734209.

[3] Emad Alsyed, Rhodri Smith, Lee Bartley, Christopher Marshall, and Emiliano Spezi. "A heterogeneous phantom study for investigating the stability of PET images radiomic features with varying reconstruction settings". In: *Frontiers in Nuclear Medicine* 3 (Feb. 2023).

[4] Judit Lantos, Erik S Mittra, Craig S Levin, and Andrei Iagarus. "Standard OSEM vs. regularized PET image reconstruction: qualitative and quantitative comparison using phantom data and various clinical radiopharmaceuticals". In: *American Journal of Nuclear Medicine and Molecular Imaging* 8 (2 2018), pp. 110–118.
